# Association between prediabetes and cognitive function in Parkinson's disease

**DOI:** 10.1002/brb3.2838

**Published:** 2022-11-30

**Authors:** Joah Park, Seohee Choi, Ryul Kim

**Affiliations:** ^1^ Department of Medicine Inha University College of Medicine Incheon Korea; ^2^ Department of Neurology Inha University Hospital, Inha University College of Medicine Incheon Korea

**Keywords:** cognition, diabetes, Parkinson's disease, prediabetes

## Abstract

**Introduction:**

It remains largely unknown whether prediabetes is related to cognitive impairment in Parkinson's disease (PD). This study aimed to assess the association between prediabetes and cognitive function in PD patients.

**Methods:**

In this cross‐sectional study, 262 PD patients (age, 69.8 ± 10.3 years; Hoehn–Yahr stage, 2.3 ± 0.8) were classified into diabetes (glycated hemoglobin [HbA1c] ≥6.5% or previously diagnosed, *n* = 76), prediabetes (5.7%–6.4%, *n* = 90), or diabetes free (≤5.6%, *n* = 96) groups. Cognitive function was measured using the Montreal Cognitive Assessment (MoCA) test.

**Results:**

Both the diabetes and prediabetes groups had significantly lower MoCA scores (17.0 ± 6.6 and 18.0 ± 6.1, respectively) than the diabetes free group (20.0 ± 5.7), even after adjusting for potential confounders (*p* = .002 and *p* = .008, respectively). In the combined group of prediabetes and diabetes free patients, higher HbA1c levels significantly correlated with lower MoCA scores (*p* = .031). There was a significant interaction of diabetes status with age, but not with the duration of PD, on cognitive function.

**Conclusion:**

In addition to diabetes, prediabetes may negatively affect cognitive function in PD patients. Further prospective longitudinal studies are necessary to clarify the impact of prediabetes on the cognitive trajectory of these patients.

## Graphical abstract

In addition to diabetes, prediabetes was associated with poorer cognitive function in PD patients, even after adjusting for multiple confounding factors. This observation was supported by a significant negative correlation between HbA1c levels and cognitive performance in PD patients without diabetes. The impact of diabetes status on cognition was influenced by age, but not PD duration.

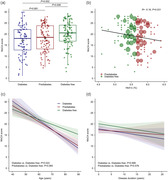

## 1 INTRODUCTION

Cognitive impairment is a common and devastating non‐motor symptom of Parkinson's disease (PD) (Aarsland et al., 2021). The prevalence of this symptom increases as the disease progresses (Aarsland et al., 2009; Hely et al., 2008). Previous longitudinal studies have shown that mild cognitive deficits affect approximately 20% of patients with early PD (Aarsland et al., 2009), and more than 80% of patients with PD developed dementia over 20 years (Hely et al., 2008). Cognitive impairment is a well‐known cause of significant functional disability and poor health‐related quality of life in PD patients (Aarsland et al., 2021).

A growing body of evidence suggests that type 2 diabetes is associated with an increased risk for PD development (Chohan et al., 2021; Komici et al., 2021; Rhee et al., 2020), accelerating disease progression, including motor and cognitive impairment (Chohan et al., 2021; Komici et al., 2021; Pagano et al., 2018). Although the exact mechanisms underlying the effect of diabetes in PD remain unclear, patients with diabetes may be susceptible to damage of nigrostriatal dopaminergic neurons, mainly due to accelerated accumulation of α‐synuclein associated with insulin resistance (Horvath & Wittung‐Stafshede, 2016).

Prediabetes is a high‐risk state for developing diabetes (Tabák et al., 2012). Emerging data provide insight into the mechanisms linking prediabetes to faster cognitive decline in dementia‐free older adults (Marseglia et al., 2019; Sundermann et al., 2021). Recent epidemiological studies have found that prediabetes is also a risk factor for subsequent PD development (Rhee et al., 2020; Sánchez‐Gómez et al., 2021). Furthermore, high non‐diabetic glycated hemoglobin (HbA1c) levels were reportedly linked to unfavorable motor outcomes in PD patients (Markaki et al., 2021). However, it remains largely unknown whether prediabetes is related to cognitive impairment in PD. Therefore, the main aim of this study was to explore the association between prediabetes and cognitive function in PD patients.

## 2 MATERIALS AND METHODS

### 2.1 Participants and clinical assessment

We retrospectively reviewed the medical records of PD patients who visited the Department of Neurology at Inha University Hospital between January 2020 and December 2021. Our general protocol for the assessment of PD in the outpatient clinic includes using the Hoehn–Yahr (H&Y) stage to evaluate motor severity, the Korean version of the Montreal Cognitive Assessment (MoCA) test to evaluate overall cognitive function, and laboratory tests including HbA1c. These measurements are generally performed without discontinuation of antiparkinsonian medications at the first visit and annually thereafter. For this study, we considered PD patients who underwent both the MoCA and HbA1c tests within a period of 1 month. PD diagnosis was based on the United Kingdom PD Brain Bank criteria. We used the first available MoCA scores and HbA1c measurement from the period during which follow‐up data existed. Patients were excluded if they were at H&Y stage 5 or if they had a history of stroke or severe white‐matter changes (grade III) (Fazekas et al., 1987). They were then categorized into three groups according to the American Diabetes Association criteria for the diagnosis of diabetes: diabetes group (HbA1c ≥ 6.5%), prediabetes group (5.7%−6.4%), and diabetes free group (≤ 5.6%) (American Diabetes, 2010). Patients who had been diagnosed with diabetes (self‐reported clinician‐diagnosed diabetes) were classified into the diabetes group, irrespective of the HbA1c level. We also collected data on age, sex, age at PD onset, PD duration, educational level, and levodopa daily equivalent dose (LEDD) for all included patients.

The study design was approved by the Institutional Review Board of Inha University Hospital (2022‐05‐025), and the need for obtaining informed consent was waived due to the retrospective nature of the study.

### 2.2 Statistical analysis

Statistical analyses were performed using *R* version 4.0.1 (R Foundation for Statistical Computing, Vienna, Austria). We conducted the Shapiro–Wilk test to test the normality of the data. Data are presented as mean, standard deviation, and frequency. Demographic and clinical characteristics were compared using one‐way analysis of variance, Kruskal–Wallis test, or chi‐square test, as appropriate. Scores on the MoCA were compared using analysis of covariance (ANCOVA), adjusted for age, sex, PD duration, educational level, H&Y stage, and LEDD. The correlation between HbA1c levels and MoCA scores was assessed using Pearson's correlation coefficient. In this analysis, we excluded the diabetes group, considering that anti‐diabetic drugs can affect HbA1c levels. We used linear regression models to evaluate the interactive associations of diabetes status with age and PD duration. The covariate terms in these models were identical to those applied in the ANCOVA. All *p* values were two‐sided, and a *p* value of less than .05 was considered statistically significant.

## 3 RESULTS

A total of 262 PD patients were included. The mean age and H&Y stage of the patients were 69.8 ± 10.3 years and 2.3 ± 0.8, respectively, and 132 (50%) were men. Further, 76 of them (29%) had diabetes, 90 (34%) had prediabetes, and 96 (37%) were diabetes free. The baseline characteristics of each group are summarized in Table 1. There were no significant differences between the groups regarding age at enrollment, sex, age at PD onset, PD duration, educational level, H&Y stage, or LEDD. The mean HbA1c levels were 6.8 ± 0.9% in the diabetes group, 6.0 ± 0.3% in the prediabetes group, and 5.4 ± 0.2% in the diabetes free group.

**TABLE 1:** Baseline characteristics

Variables	Total	Diabetes	Prediabetes	Diabetes free	*p* Value
Number of subjects	262	76	90	96	—
Age, years	69.8 (10.3)	71.1 (8.0)	69.2 (8.3)	69.8 (10.3)	.397
Male sex, %	132 (50%)	43 (57%)	40 (44%)	49 (51%)	.293
Age at PD onset, years	64.9 (9.2)	65.2 (8.6)	64.4 (8.4)	65.2 (10.3)	.810
PD duration, years	5.1 (4.7)	5.9 (5.4)	4.8 (4.5)	4.7 (4.4)	.174
Educational level, years	9.8 (5.5)	9.2 (6.3)	9.2 (5.2)	10.8 (5.1)	.092
Hoehn and Yahr stage	2.3 (0.8)	2.5 (0.8)	2.3 (0.8)	2.3 (0.7)	.126
LEDD, mg	521.7 (337.0)	568.6 (377.4)	498.9 (316.0)	505.9 (321.6)	.351
HbA1c level, %	6.0 (0.8)	6.8 (0.9)	6.0 (0.3)	5.4 (0.2)	<.001

*Note*: Data are presented as *n* (%) or mean (standard deviation).

Abbreviations: HbA1c, glycated hemoglobin; LEDD, levodopa equivalent daily dose; PD, Parkinson's disease.

**FIGURE 1:**
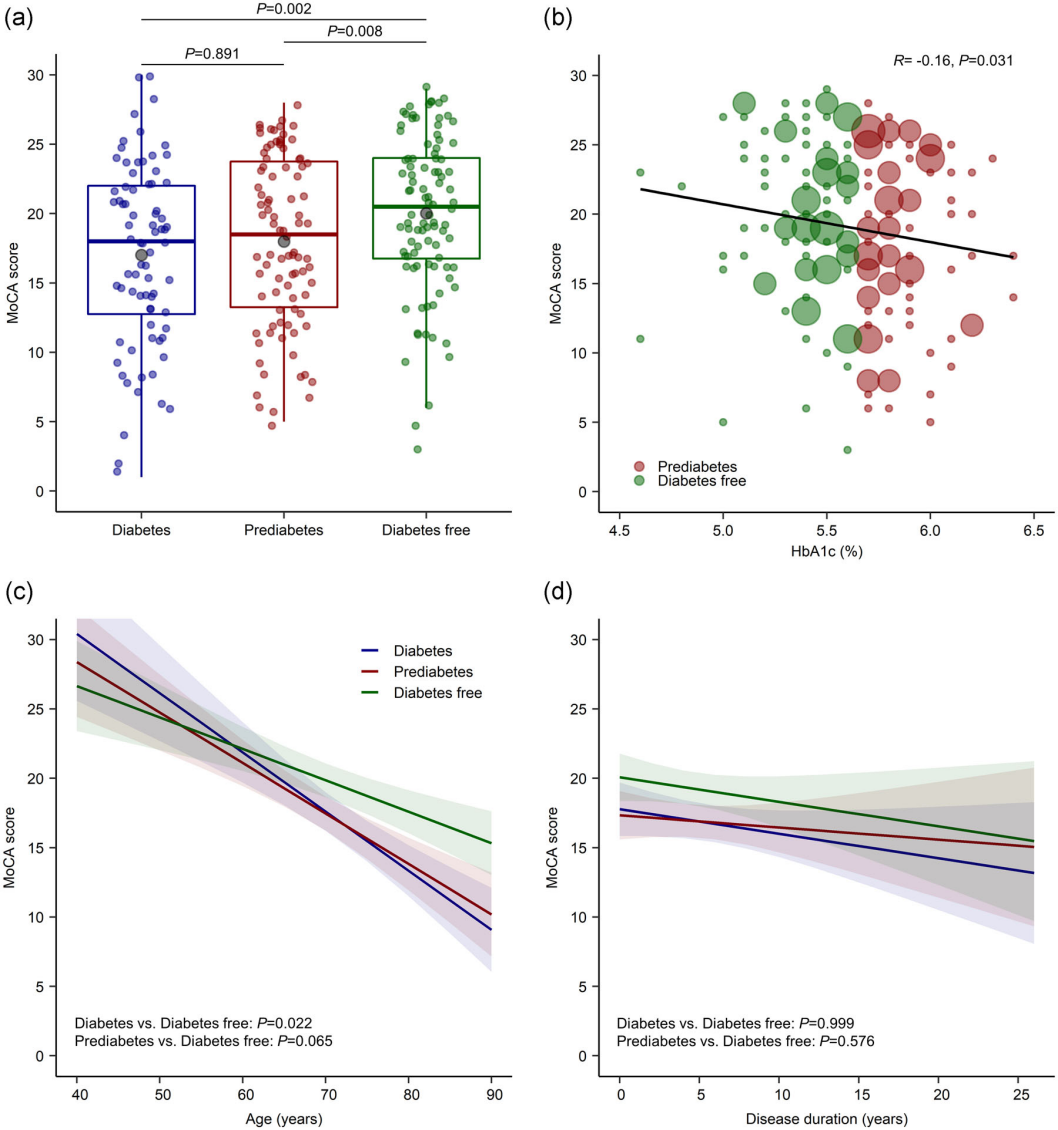
Association of MoCA scores with diabetes status in patients with PD. (a) The boxes represent interquartile ranges, with the horizontal line in each box representing the median and the whiskers showing the minimum and maximum values (excluding outliers that were more than 1.5 times the values represented at each end of the box). The grey circles represent the mean values. (b) Linear correlation between MoCA scores and HbA1c levels in non‐diabetic patients with PD. The size of the plotted symbol is proportional to the number of observations. (c,d) Interactive associations of MoCA scores with age (c) and disease duration (d). Shaded regions indicate 95% confidence intervals. HbA1c, glycated hemoglobin; MoCA, Montreal Cognitive Assessment; PD, Parkinson's disease

The MoCA scores in the diabetes, prediabetes, and diabetes free groups were 17.0 ± 6.6, 18.0 ± 6.1, and 20.0 ± 5.7, respectively. After adjusting for covariates, we found that both the diabetes and prediabetes groups had significantly lower MoCA scores than the diabetes free group (*p* = .002 and *p* = .008, respectively), although there was a considerable overlap in the scores between the three groups (shown in Figure 1a). Performance on the MoCA test did not differ significantly between the diabetes and prediabetes groups (*p* = .891). Next, we examined the correlation between HbA1c levels and MoCA scores, regardless of the disease state. Higher HbA1c levels were correlated with lower MoCA scores in the prediabetes and diabetes free groups (*R* = −.16, *p* = .031) (shown in Figure 1b).

In the linear regression models adjusted for covariates, the diabetes group had a steeper rate of cognitive decline with aging than the diabetes free group (*p* = .022) (shown in Figure 1c). Similarly, the rate of cognitive decline tended to be steeper with age in the prediabetes group compared to the diabetes free group, although the difference was not statistically significant (*p* = .065). There were no significant interactive effects between diabetes status and the duration of PD shown in Figure 1d.

## 4 DISCUSSION

The main finding of this study is that, besides diabetes, prediabetes was associated with poorer cognitive function in PD patients, even after adjusting for multiple confounding factors. This observation was supported by a significant negative correlation between HbA1c levels and cognitive performance in PD patients without diabetes. However, the difference in MoCA scores according to diabetes status was modest, indicating that the difference was statistically significant but may not be clinically meaningful. Notably, the impact of diabetes status on cognition was influenced by age, but not PD duration.

There are several possible explanations for the association between hyperglycemia and lower cognitive performance in PD patients. It has been suggested that insulin resistance, a prominent common feature of diabetes and prediabetes, not only results in elevation of islet amyloid polypeptide, but also inhibits insulin‐degrading enzymes (Cheong et al., 2020; Horvath & Wittung‐Stafshede, 2016). A growing body of evidence suggests that these changes can promote nigrostriatal dopaminergic degeneration and the aggregation of cortical α‐synuclein (Morris et al., 2011; Sun et al., 2020), which may have contributed to the observed findings. Additionally, both diabetes and prediabetes have been reported to cause oxidative stress and enhanced neuroinflammation (Luc et al., 2019), which are regarded as underlying pathomechanisms in PD dementia (Aarsland et al., 2021). Although there was no significant interaction between prediabetes and PD duration affecting cognition, we could not access data regarding the duration of prediabetes in this cross‐sectional dataset, and thus, their interaction still remains unclear. However, we did not find a significant interaction between diabetes and PD duration as well. Given that diabetes is generally preceded by a long period of prediabetic status (Tabák et al., 2012), the above PD‐specific explanations may not be supported, at least for patients in the prediabetic group. Furthermore, there were no differences in H&Y stage and LEDD between the groups, suggesting that the impact of nigrostriatal dopaminergic pathway is less likely to be significant in our findings.

Alternatively, it is possible that mechanisms that cause prediabetes to negatively affect cognitive functions are not PD‐specific, as suggested by our finding that the relationship between diabetes status and cognitive function is affected by age itself. In this context, one plausible explanation is that age‐related Alzheimer's pathologies, mainly characterized by the deposition of amyloid and tau in the brain, are accelerated via hyperglycemic status (Ramos‐Rodriguez et al., 2017). Moreover, it is known that the presence of diabetes and prediabetes provokes microstructural white matter abnormalities (Marseglia et al., 2019), and these abnormalities are linked to worse cognitive function in PD patients (Aarsland et al., 2021).

The present study has some limitations. First, because this study was designed to be cross‐sectional, we collected data at a single point in time and, therefore, could not determine the causality of cognitive impairment related to prediabetes. Second, although some anti‐diabetic drugs, particularly metformin and glucagon‐like peptide‐1 receptor agonists, may have beneficial effects on cognitive function in PD patients (Chen et al., 2021), we could not access each patient's medications in the diabetes group due to lack of data. Thus, the effect of diabetes on cognition in our dataset may have been underestimated. Nevertheless, our results show that the presence of prediabetes and diabetes may negatively influence cognitive function in PD patients. Further prospective longitudinal studies are warranted to clarify the impact of prediabetes on the cognitive trajectory of these patients.

## AUTHOR CONTRIBUTIONS

Joah Park and Ryul Kim participated in the study design, data collection, data analysis, and data interpretation. Seohee Choi participated in the data collection and data analysis. All authors provided critical review of the manuscript and approved the final draft.

## CONFLICT OF INTEREST

The authors have no conflict of interest.

### PEER REVIEW

The peer review history for this article is available at https://publons.com/publon/10.1002/brb3.2838.

## Data Availability

The dataset supporting the conclusions of this article is included within the article. Further enquiries can be directed to the corresponding author.

## References

[brb32838-bib-0001] Aarsland, D. , Batzu, L. , Halliday, G. M. , Geurtsen, G. J. , Ballard, C. , Ray Chaudhuri, K. , & Weintraub, D. (2021). Parkinson disease‐associated cognitive impairment. Nature Reviews Disease Primers, 7(1), 47. 10.1038/s41572-021-00280-3 34210995

[brb32838-bib-0002] Aarsland, D. , Brønnick, K. , Larsen, J. P. , Tysnes, O. B. , & Alves, G. (2009). Cognitive impairment in incident, untreated parkinson disease: The Norwegian Parkwest study. Neurology, 72(13), 1121–1126. 19020293 10.1212/01.wnl.0000338632.00552.cb

[brb32838-bib-0003] American Diabetes, A. (2010). Standards of medical care in diabetes—2010. Diabetes Care, 33(1), S11–S61. 20042772 10.2337/dc10-S011 PMC2797382

[brb32838-bib-0004] Chen, Q. , Cao, T. , Li, N. , Zeng, C. , Zhang, S. , Wu, X. , Zhang, B. , & Cai, H. (2021). Repurposing of anti‐diabetic agents as a new opportunity to alleviate cognitive impairment in neurodegenerative and neuropsychiatric disorders. Frontiers in Pharmacology, 12, 667874. 34108878 10.3389/fphar.2021.667874 PMC8182376

[brb32838-bib-0005] Cheong, J. L. Y. , de Pablo‐Fernandez, E. , Foltynie, T. , & Noyce, A. J. (2020). The association between type 2 diabetes mellitus and Parkinson's disease. Journal of Parkinson's Disease, 10(3), 775–789. 10.3233/JPD-191900 PMC7458510 32333549

[brb32838-bib-0006] Chohan, H. , Senkevich, K. , Patel, R. K. , Bestwick, J. P. , Jacobs, B. M. , Ciga, S. B. , Gan‐Or, Z. , & Noyce, A. J. (2021). Type 2 diabetes as a determinant of Parkinson's disease risk and progression. Movement Disorders, 36(6), 1420–1429. 33682937 10.1002/mds.28551 PMC9017318

[brb32838-bib-0007] Fazekas, F. , Chawluk, J. B. , Alavi, A. , Hurtig, H. I. , & Zimmerman, R. A. (1987). MR signal abnormalities at 1.5 t in Alzheimer's dementia and normal aging. American Journal of Roentgenology, 149(2), 351–356. 3496763 10.2214/ajr.149.2.351

[brb32838-bib-0008] Hely, M. A. , Reid, W. G. , Adena, M. A. , Halliday, G. M. , & Morris, J. G. (2008). The sydney multicenter study of Parkinson's disease: The inevitability of dementia at 20 years. Movement Disorders, 23(6), 837–844. 18307261 10.1002/mds.21956

[brb32838-bib-0009] Horvath, I. , & Wittung‐Stafshede, P. (2016). Cross‐talk between amyloidogenic proteins in type‐2 diabetes and Parkinson's disease. Proceedings of the National Academy of Sciences, 113(44), 12473–12477. 10.1073/pnas.1610371113 PMC5098634 27791129

[brb32838-bib-0010] Komici, K. , Femminella, G. D. , Bencivenga, L. , Rengo, G. , & Pagano, G. (2021). Diabetes mellitus and Parkinson's disease: A systematic review and meta‐analyses. Journal of Parkinson's Disease, 11(4), 1585–1596. 10.3233/JPD-212725 34486987

[brb32838-bib-0011] Luc, K. , Schramm‐Luc, A. , Guzik, T. J. , & Mikolajczyk, T. P. (2019). Oxidative stress and inflammatory markers in prediabetes and diabetes. Journal of Physiology and Pharmacology, 70(6), 809–824. 10.26402/jpp.2019.6.01 32084643

[brb32838-bib-0012] Markaki, I. , Ntetsika, T. , Sorjonen, K. , & Svenningsson, P. (2021). Euglycemia indicates favorable motor outcome in Parkinson's disease. Movement Disorders, 36(6), 1430–1434. 33634916 10.1002/mds.28545

[brb32838-bib-0013] Marseglia, A. , Fratiglioni, L. , Kalpouzos, G. , Wang, R. , Bäckman, L. , & Xu, W. (2019). Prediabetes and diabetes accelerate cognitive decline and predict microvascular lesions: A population‐based cohort study. Alzheimers & Dementia, 15(1), 25–33. 10.1016/j.jalz.2018.06.3060 30114414

[brb32838-bib-0014] Morris, J. K. , Bomhoff, G. L. , Gorres, B. K. , Davis, V. A. , Kim, J. , Lee, P. P. , Brooks, W. M. , Gerhardt, G. A. , Geiger, P. C. , & Stanford, J. A. (2011). Insulin resistance impairs nigrostriatal dopamine function. Experimental Neurology, 231(1), 171–180. 21703262 10.1016/j.expneurol.2011.06.005 PMC3169014

[brb32838-bib-0015] Pagano, G. , Polychronis, S. , Wilson, H. , Giordano, B. , Ferrara, N. , Niccolini, F. , & Politis, M. (2018). Diabetes mellitus and Parkinson disease. Neurology, 90(19), e1654–e1662. 29626177 10.1212/WNL.0000000000005475

[brb32838-bib-0016] Ramos‐Rodriguez, J. J. , Spires‐Jones, T. , Pooler, A. M. , Lechuga‐Sancho, A. M. , Bacskai, B. J. , & Garcia‐Alloza, M. (2017). Progressive neuronal pathology and synaptic loss induced by prediabetes and type 2 diabetes in a mouse model of Alzheimer's disease. Molecular Neurobiology, 54(5), 3428–3438. 27177549 10.1007/s12035-016-9921-3

[brb32838-bib-0017] Rhee, S. Y. , Han, K. D. , Kwon, H. , Park, S. E. , Park, Y. G. , Kim, Y. H. , Yoo, S. J. , Rhee, E. J. , & Lee, W. Y. (2020). Association between glycemic status and the risk of Parkinson disease: A nationwide population‐based study. Diabetes Care, 43(9), 2169–2175. 32611610 10.2337/dc19-0760 PMC7440896

[brb32838-bib-0018] Sánchez‐Gómez, A. , Díaz, Y. , Duarte‐Salles, T. , Compta, Y. , & Martí, M. J. (2021). Prediabetes, type 2 diabetes mellitus and risk of Parkinson's disease: A population‐based cohort study. Parkinsonism Related Disorders, 89, 22–27. 34216937 10.1016/j.parkreldis.2021.06.002

[brb32838-bib-0019] Sun, Y. , Guo, C. , Yuan, L. , Li, W. , Wang, Z. Y. , Yue, F. , & Li, J. Y. (2020). Cynomolgus monkeys with spontaneous type‐2‐diabetes‐mellitus‐like pathology develop alpha‐synuclein alterations reminiscent of prodromal Parkinson's disease and related diseases. Frontiers in Neuroscience, 14, 63. 32116510 10.3389/fnins.2020.00063 PMC7019001

[brb32838-bib-0020] Sundermann, E. E. , Thomas, K. R. , Bangen, K. J. , Weigand, A. J. , Eppig, J. S. , Edmonds, E. C. , Wong, C. G. , Bondi, M. W. , & Delano‐Wood, L. (2021). Prediabetes is associated with brain hypometabolism and cognitive decline in a sex‐dependent manner: A longitudinal study of nondemented older adults. Frontiers in Neurology, 12, 551975. 33679574 10.3389/fneur.2021.551975 PMC7933503

[brb32838-bib-0021] Tabák, A. G. , Herder, C. , Rathmann, W. , Brunner, E. J. , & Kivimäki, M. (2012). Prediabetes: A high‐risk state for diabetes development. Lancet, 379(9833), 2279–2290. 22683128 10.1016/S0140-6736(12)60283-9 PMC3891203

